# The value of CT radiomic in differentiating mycoplasma pneumoniae pneumonia from streptococcus pneumoniae pneumonia with similar consolidation in children under 5 years

**DOI:** 10.3389/fped.2022.953399

**Published:** 2022-09-28

**Authors:** Dongdong Wang, Jianshe Zhao, Ran Zhang, Qinghu Yan, Lu Zhou, Xiaoyu Han, Yafei Qi, Dexin Yu

**Affiliations:** ^1^Department of Radiology, Qilu Hospital, Cheeloo College of Medicine, Shandong University, Jinan, China; ^2^Department of Radiology, Children’s Hospital Affiliated to Shandong University, Jinan, China; ^3^Huiying Medical Technology (Beijing) Co., Ltd., Beijing, China; ^4^Department of Ultrasound, Shandong Public Health Clinical Center, Jinan, China; ^5^Department of Cardiac Surgery ICU, Qilu Hospital of Shandong University, Jinan, China

**Keywords:** pneumonia, mycoplasma pneumoniae pneumonia, streptococcus pneumoniae pneumonia, nomogram, CT radiomics

## Abstract

**Objective:**

To investigate the value of CT radiomics in the differentiation of mycoplasma pneumoniae pneumonia (MPP) from streptococcus pneumoniae pneumonia (SPP) with similar CT manifestations in children under 5 years.

**Methods:**

A total of 102 children with MPP (*n* = 52) or SPP (*n* = 50) with similar consolidation and surrounding halo on CT images in Qilu Hospital and Qilu Children’s Hospital between January 2017 and March 2022 were enrolled in the retrospective study. Radiomic features of the both lesions on plain CT images were extracted including the consolidation part of the pneumonia or both consolidation and surrounding halo area which were respectively delineated at region of interest (ROI) areas on the maximum axial image. The training cohort (*n* = 71) and the validation cohort (*n* = 31) were established by stratified random sampling at a ratio of 7:3. By means of variance threshold, the effective radiomics features, SelectKBest and least absolute shrinkage and selection operator (LASSO) regression method were employed for feature selection and combined to calculate the radiomics score (Rad-score). Six classifiers, including k-nearest neighbor (KNN), support vector machine (SVM), extreme gradient boosting (XGBoost), random forest (RF), logistic regression (LR), and decision tree (DT) were used to construct the models based on radiomic features. The diagnostic performance of these models and the radiomic nomogram was estimated and compared using the area under the receiver operating characteristic (ROC) curve (AUC), and the decision curve analysis (DCA) was used to evaluate which model achieved the most net benefit.

**Results:**

RF outperformed other classifiers and was selected as the backbone in the classifier with the consolidation + the surrounding halo was taken as ROI to differentiate MPP from SPP in validation cohort. The AUC value of MPP in validation cohort was 0.822, the sensitivity and specificity were 0.81 and 0.81, respectively.

**Conclusion:**

The RF model has the best classification efficiency in the identification of MPP from SPP in children, and the ROI with both consolidation and surrounding halo is most suitable for the delineation.

## Introduction

Pneumonia is the leading cause of death among children under 5 years of age worldwide (1–3). Mycoplasma pneumoniae pneumonia (MPP) and Streptococcus pneumoniae pneumonia (SPP) are common types of pneumonia in children (4, 5). Consolidation and lung abscesses caused by pulmonary pathogens can present as poor efficacy of antimicrobial therapy in the acute stage, and relate with complications including pleural effusion, necrotizing pneumonia, and even higher mortality. Early identification of the etiology and corresponding treatment can significantly reduce the mortality rate, however, reliable samples of the biological causes of childhood pneumonia are difficult to obtain in clinical practice (6). In addition, the colonization of pathogenic microorganisms in upper respiratory tract samples (7), nasal wipes, oropharyngeal wipes or sputum detection cannot accurately reflect the infection of the lower respiratory tract (8, 9). At the same time, invasive lung puncture biospy, bronchoalveolar lavage, and other techniques cannot be used as routine detection methods in children. Currently, common pathogen detection methods have many defects such as long detection cycle time, false positive, and false negative results (10–13), which are greatly limited in practical clinical applications. Meanwhile, the imaging manifestations of MPP and SPP in most cases are similar in practical clinical situation, leading to difficulties in the differential diagnosis. Thus the timely and effective treatment requires accurate etiological details (14).

In recent years, the radiomics analysis based on massive data and artificial intelligence has shown significant advantages in judging disease types, predicting risk, and guiding treatment (15–17). Radiomics converts medical images into high-dimensional images and mines effective data features through quantitative high-throughput extraction for data analysis, so various information that cannot be identified by the naked eye of radiologists, such as texture features, can be extracted, which is helpful for the diagnosis and treatment of diseases (18). At the same time, this technology is simple and quick, and has a potential to solve the identification problems between both the pneumonias. It is especially suitable for the patients who cannot obtain the results of pathogen detection in a short period of time, but are in critical condition and need accurate medication urgently.

We speculated that radiomics may be able to find more information that is not visible to the naked eye and may facilitate the differentiation of these two types of diseases. In this study, we collected a group of pediatric MPP and SPP patients with similar CT manifestations and difficulty in visual differentiation to investigate the value of CT radiomics in the differentiation of MPP and SPP. To the best of our knowledge, this is the first study to investigate the identification of pneumonia in children by radiomics.

## Materials and methods

### Study design

This study was a retrospective cohort study.

### Data source and collection

This study was approved by the Ethics Committee of Qilu Hospital of Shandong University and Qilu Children’s Hospital. Children with MPP and SPP who were admitted to Qilu Hospital of Shandong University or Qilu Children’s Hospital on January 1, 2017 and July 3, 2022 were collected. All cases were diagnosed by clinical features and nucleic acid detection from bronchoalveolar lavage fluid. The chest CT images of the enrolled children were retrospectively analyzed. All the children were consistent with the clinical manifestations of pneumonia, such as fever, cough, and the presence of corresponding imaging findings. Then, 102 cases of pediatric pneumonia patients enrolled in the study were stratified and randomly sampled in a ratio of 7:3. All patients were divided into the training cohort (71 cases) and the validation cohort (31 cases).

Inclusion criteria: (1) Bronchoscopy was collected for alveolar lavage fluid for multiplex polymerase chain tests reaction (mPCR) detection in all cases. Mycoplasma infection and Streptococcus pneumoniae infection were confirmed, and the corresponding treatment was effective. (2) Mycoplasma detection by a particle agglutination test, where the single MP-IgM antibody titer was ≥1:160, or the MP-IgM antibody titer in the recovery and acute phases was four or more times higher than initial results. (3) Streptococcus diagnosed by the culture of sputum. (4) CT images showed predominantly solid lesions on the lung window. (5) Two experienced associate professors in chest imaging diagnosis were unable to identify the nature of pneumonia. Exclusion criteria: (1) CT images have motion artifacts, poor image quality, large differences in scanning conditions, and inconsistent slice thickness; (2) There is clinical suspicion of mixed infection.

### Sampling procedures

A pediatric flexible fiberoptic bronchoscope was inserted through the mouth to avoid nasal contamination. Aliquots of saline solution (0.9%) were dripped into the diseased lobar or segmental bronchus (maximum volume, 3 mL/kg body weight). The first bronchial lavage fraction was discarded. The same amount of 4% NaOH was added, and the mixture was fully stirred and liquefied at 37^°^C for 30 min. Fifty to hundred microliter nucleic acid extract was added to perform respiratory mPCR with the DNA and Viral Nucleic Acid Volume kit according to manufacturer’s instructions. All subjects were sedated with intravenous midazolam, atropinesulphate, and tramadol hydrochloride. Percutaneous monitoring of oxygen saturation and heart rate; during the operation, oxygen was provided as needed.

Induced sputum specimens were collected from individuals aged ≥3 months. If induced sputum collection was contraindicated or was not advised by the attending clinician and a patient was able to expectorate, expectorated sputum was collected. Serological tests were performed by the complement fixation technique.

### CT scanning method

Non-inspection parts are coated with lead to reduce the radiation damage to children. SOMATOM Definition AS 64-slice spiral CT scanner was used to perform conventional chest scanning with the range from lung tip to the upper abdomen level of 5 cm below the diaphragmatic dome. The scanning parametric were as follows: tube voltage 120 KV, tube current 250∼400 mA/s (using automatic tube current modulation), FOV: 18∼35 cm, matrix 512 × 512, slice thickness 5 mm, slice spacing 5 mm, scanning time 1.0 s. Patients who did not coordinate with the examination were routinely given sedative drugs.

### Image delineation

**FIGURE 1:**
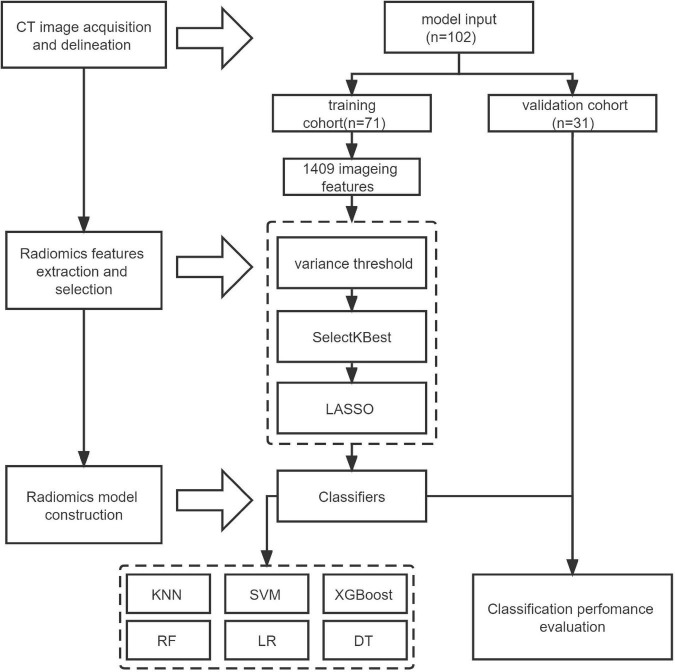
Flowchart of the whole radiomics study.

**FIGURE 2:**
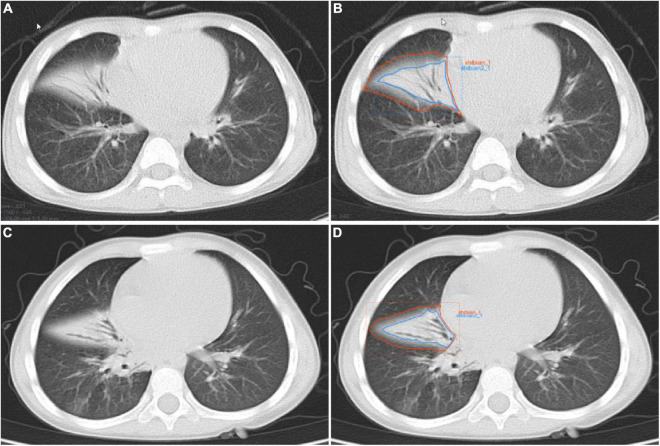
Manual delineation on lung window CT images in a 25-month female patient with mycoplasma pneumoniae pneumonia **(A,B)** and a 21-month male patient with streptococcus pneumoniae pneumonia **(C,D)**. CT shows the similar appearances with consolidation and surrounding halo in middle lobe of right lung **(A,C)**, and two ROIs (blue line and orange line) are delineated in each patient.

The flow-chart depicting image of feature extraction and selection and model construction is presented in Figure 1. The ROIs of the lesions on all lung window CT images were assessed and delineated in a double-blind manner by two radiologists with 5 and 10 years of experience, respectively, and following review was performed by a senior physician. If the difference was ≥5%, the latter would determine the boundary and redraw it. Two different kinds of ROI delineation of the lesion were made (Figures 2A–D): the first included the single consolidation part of the lesion (Figures 2B,D, blue line) and the second included the consolidation and surrounding halo area (Figures 2B,D, orange line). Meanwhile, the mediastinal window images was also used for the judgment of consolidation part of the lesion as the reference. The cavity, necrosis, hemorrhage or ground glassin oppacities in lesion were also included in the ROI. At the same time, the adjacent mediastinum, thickening pleura, and pleural effusion were avoided to draw by referring to these structures on mediastinal window CT. Grayscale normalization was then performed to reduce the impact of contrast and brightness changes. Ultimately, 102 ROIs were segmented from CT images of 102 patients and used for subject analysis.

### Feature extraction and selection

A total of 1,409 quantitative image features were extracted from CT images using a RadCloud platform.^1^ These properties were divided into three groups. The first group (first order statistics) consisted of 126 descriptors that could quantitatively describe the distribution of voxel intensities on CT images through commonly used basic indicators. The second group (shape- and size-based features) reflecting the shape and size of the region. According to the operation length of gray level and the calculation of gray level co-occurrence texture matrix, 525 texture features that could quantify regional heterogeneity differences were classified into three categories (texture features). Texture features then characterized the recurrent local patterns on the image with their arrangement rules, including 75 features Gray Level Co-occurrence Matrix (GLCM), Gray Level Run Length Matrix (GLRLM) and Gray Level Size Zone Matrix (GLSZM). In addition, the texture was multi-resolution represented by filtering the image using 14 filters including index, logarithm, gradient, square value, square root, lbp-2D and wavelet (Wavelet-LHL, Wavelet-LHH, Wavelet-HLL, Wavelet-LLH, Wavelet-HLH, Wavelet-HHH, Wavelet-HHL, and Wavelet-LLL) to analyze the texture on a finer scale.

For intra-observer and inter-observer variation, intra-observer and inter-observer consistency of each feature was quantified by intra-class correlation (ICC) between calculated feature pairs, features with low reproducibility were excluded from subsequent analysis, and any feature with an ICC less than 0.8 was discarded. Redundant features could be reduced by reducing and selecting features to obtain the best results. The feature selection method used variance threshold (variance threshold = 0.8), SelectKBest and LASSO models. For the variance threshold method, the threshold was 0.8, so that the eight values of the variance smaller than 0.8 were removed. The SelectKBest method belonged to the univariate feature selection method, which used *p*-values to analyze the relationship between features and classification results, and all features with *p*-values less than 0.05 would be used. For the LASSO model, the L1 regularizer was used as the cost function, the error value of cross-validation was 5, and the maximum number of iterations was 1,000.

### Radiomics model construction

Based on the clinical data and the follow-up imaging analysis, the training and validation cohorts were stratified and randomly sampled at a ratio of 7:3 to establish the training cohort (*n* = 71) and the validation cohort (*n* = 31), and the number of random seeds was 734. Six classifiers, including KNN, SVM, XGBoost, RF, LR, and DT, were used to construct an Radiomics-based machine learning model to model MPP with SPP.

### Statistical analysis

R software (v. 3.5.1) and SPSS26.0 were used to perform statistical analysis of the data. Age difference in both diseases was tested by independent sample *t*-test. Chi square test was used to analyze the differences in gender and inflammatory site. The linear combination of the selected features and the product of the corresponding weighting coefficients was used to form the radiomics label for each patient. The nomogram construction and calibration plotting were used by the “rms” package. The decision curve analysis (DCA) curve plots were performed using the “rmda” package. ROC analysis was used to evaluate the diagnostic performances of the classifiers, and the accuracy (score) matrix was established to compare and evaluate different radiomics models. *P* < 0.05 was considered to indicate statistical significance.

## Results

### Study population

A total of 102 children were enrolled in this study, including 52 children with mycoplasma infection, with an average age of 33.06 ± 14.34 months. The mean age of 50 patients with Streptococcus pneumoniae was 30.9 ± 15.4 months. There were no significant differences in age and gender between MPP and SPP patients (*P* > 0.05), as shown in Table 1.

**TABLE 1:** Comparison of patients’ general information.

Characteristics	Training cohort	*P*	Validation cohort	*P*
		
	MPP (*n* = 36) SPP (*n* = 35)		MPP (*n* = 16) SPP (*n* = 15)	
Age (month)	35.3333 ± 14.25683	0.268	27.9375 ± 14.99986	0.794
	31.4857 ± 14.74762		29.4667 ± 17.21240	
Gender, *n* (%)		0.111		0.289
Male	42 (59.2%)		16 (51.6%)	
Female	29 (40.8%)		15 (48.4%)	

### Feature extraction and screening results

**FIGURE 3:**
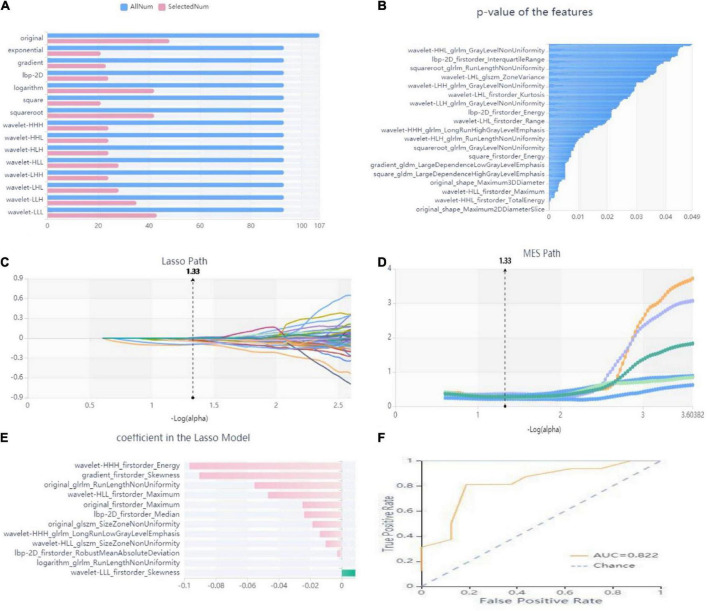
Workflow model construction and radiomics analysis. **(A)** A variance threshold on feature select. The blue bar represents the number of all the extracted radiomics features, and the pink bar represents the number of radiomics features screened by variance threshold method. The vertical axis is 15 kinds of filtering methods (variance threshold = 0.8). **(B)** SelectKBest on feature select. The abscissa is the *P*-value of the feature, and the ordinate is the feature whose *P* value < 0.05 is screened by SelectKBest method. **(C–E)** Schematic diagram of feature screening by Lasso method: **(C)** Lasso path, where the abscissa is the log value of α, and the ordinate is the coefficient of the feature. **(D)** The abscissa of the MSE path is the log value of α, and the ordinate is the mean square error. **(E)** Regression coefficient of Lasso model, where the abscissa represents the regression coefficient and the ordinate represents the selected features. **(F)** ROC curve of the RF model. Yellow curve is MPP cohort, blue curve is SPP cohort.

In this study, we firstly select 451 features from 1,409 features using variance threshold method (Figure 3A), then 151 features were screened out by SelectKBest methods (Figure 3B), and finally 12 optimal features were screened out by LASSO algorithm (Figures 3C–E). RF outperformed other classifiers and was selected as the backbone in the classifier with the consolidation + the surrounding halo was taken as ROI to differentiate MPP from SPP in validation cohort. The radiomics analysis report with ROI in the consolidation part and surrounding halo area was shown in Supplementary material 1. The radiomics analysis report with ROI of the consolidation region was shown in Supplementary material 2.

The radiology score (Rad-score) formula was constructed based on these seven features and their regression coefficients (Table 2), and the formula was: Rad-score = wavelet-HHH_firstorder_Energy * −0.09673 + gradient_firstorder_ Skewness*−0.09053 + wavelet-HLL_firstorder_Maximum* −0.04688 + wavelet-HHH_glrlm_LongRunLowGrayLevel Emphasis*−0.0139 + lbp-2D_firstorder_Median*−0.02388 + original_glszm_SizeZoneNonUniformity*−0.0102 + wavelet- LLL_firstorder_Skewness*0.00864 + original_firstorder_ Maximum*−0.02489 + lbp-2D_firstorder_RobustMean AbsoluteDeviation*−0.00281 + original_glrlm_RunLengthNon Uniformity*−0.0554 + logarithm_glrlm_RunLengthNon Uniformity*−0.00001 + wavelet-HLL_glszm_SizeZoneNon Uniformity*−0.01856.

**TABLE 2:** Radiomics features and their categories, filters and regression coefficients selected with ROI in the consolidation cohort and surrounding halo area.

Radiomic feature	Radiomic class	Filter	Coefficient
Energy	firstorder	wavelet-HHH	–0.09673
Skewness	firstorder	Gradient	–0.09053
Maximum	firstorder	wavelet-HLL	–0.04688
LongRunLowGrayLevelEmphasis	glrlm	wavelet-HHH	–0.01390
Median	firstorder	lbp-2D	–0.02388
SizeZoneNonUniformity	glszm	Original	–0.01020
Skewness	firstorder	wavelet-LLL	0.00864
Maximum	firstorder	Original	–0.02489
RobustMeanAbsoluteDeviation	firstorder	lbp-2D	–0.00281
RunLengthNonUniformity	glrlm	Original	–0.05540
RunLengthNonUniformity	glrlm	Logarithm	–0.00001
SizeZoneNonUniformity	glszm	wavelet-HLL	–0.01856

### Differential efficacy of radiomics models

The analysis results of accuracy (score) matrix in validation cohort of six models with two kinds of ROI is shown in Table 3. All classifiers in consolidation region are shown in Table 4. After the ROI contained surrounding halo area, the matrix scores of all classifiers were significantly improved. The ROC curve analysis results of all classifier in validation cohort are shown in Table 5 and the RF classifier get the best matrix score. When this classifier was used for validation cohort, the AUC of MPP was 0.822 [95% confident interval (CI): 0.684–0.960, sensitivity = 0.81, specificity = 0.81] (Figure 3F). The four indicators of the classifier (accuracy, recall rate, F1 score, and support) are shown in Table 6.

**TABLE 3:** Accuracy (score) matrix in validation cohort of six models with two kinds of ROI.

	Validation_score of the consolidation area	Validation_score of the consolidation + surrounding halo
KNN	0.610	0.630
SVM	0.610	0.720
XGBoost	0.650	0.720
RF	0.610	0.810
LR	0.580	0.720
DT	0.520	0.690

**TABLE 4:** ROC curve analysis results in validation cohort with ROI of the consolidation region.

Classifiers	AUC	95% CI	Sensitivity	Specificity
KNN	0.581	0.404–0.758	0.600	0.630
SVM	0.533	0.356–0.710	0.530	0.690
XGBoost	0.563	0.395–0.731	0.470	0.690
RF	0.498	0.326–0.670	0.530	0.630
LR	0.575	0.406–0.744	0.400	0.690
DT	0.544	0.378–0.710	0.400	0.690

**TABLE 5:** ROC curve analysis results in validation cohort with ROI of both the consolidation cohort and surrounding halo area.

Classifiers	AUC	95% CI	Sensitivity	Specificity
KNN	0.727	0.556–0.898	0.690	0.560
SVM	0.797	0.639–0.955	0.750	0.690
XGBoost	0.785	0.622–0.948	0.440	0.750
RF	0.822	0.684–0.960	0.810	0.810
LR	0.734	0.574–0.894	0.690	0.750
DT	0.688	0.538–0.838	0.500	0.880

**TABLE 6:** Evaluation results of the four indicators of the both diseases in validation cohort with ROI of both the consolidation cohort and surrounding halo area.

Indicators	KNN	SVM	XGBoost	RF	LR	DT
Precision	0.610	0.710	0.640	0.810	0.730	0.800
Sensitivity	0.690	0.750	0.440	0.810	0.690	0.500
F1-score	0.650	0.730	0.520	0.810	0.710	0.620
Support	16	16	16	16	16	16

### Development and performance of the radiomic nomogram

**FIGURE 4:**
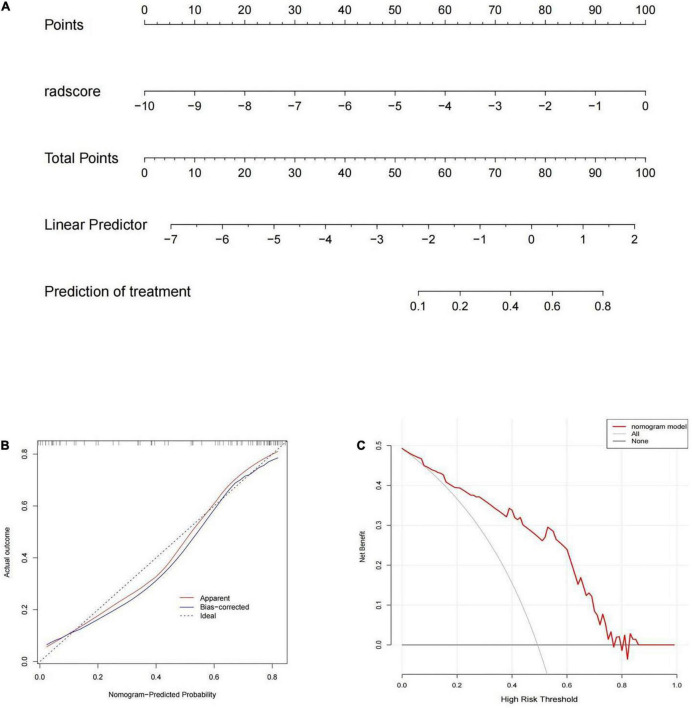
**(A)** The radiomic nomogram was built on the training group with the rad-score. **(B)** The calibration curve in the training cohort. **(C)** The decision curve analysis (DCA) curve of the radiomic nomogram in the training cohort.

The rad-score was identified as independent predictors for discriminating between MPP and SPP and then a radiomic nomogram was developed. The overall number of points for each patient was computed using the nomogram and was associated with the likelihood of MPP. Details of the performance of radiomic nomogram are shown in Figures 4A,B. Finally, a DCA was performed to evaluate whether this nomogram would assist in differentiating between MPP from SPP (Figure 4C).

## Discussion

In this retrospective study, we established six classifiers to evaluated the performance for discriminating MPP from SPP with two kinds of ROI. We found that the radiomics classifier demonstrated low performance for differentiation using the ROI of consolidation region. After the ROI contained surrounding halo area, every classifiers performed better and demonstrated high performance. Our preliminary results revealed that classifiers trained with ROI of both the consolidation part and surrounding halo area achieved better diagnostic performance for discrimination between MPP and SPP, with significantly higher AUC than the ROI of consolidation region in the validation cohort and in all patients.

The differentiation between MPP and SPP in children is pivotal, as treatment approaches are quite different (19). Accurate and early etiological diagnosis can guide clinicians in rational drug using (20), reducing the total mortality rate by 27% and the pneumonia-specific mortality rate by 42% (21). At the same time, it can also reduce the abuse of antibiotics and improve bacterial drug resistance. Therefore, the accurate diagnosis and identification is helpful in guiding treatment.

In radiomics, there are a variety of machine learning methods that can be used to build classification models, which have their own advantages for different tasks. In this study, six commonly used classifier models (KNN, SVM, XGBoost, RF, LR, and DT) and 1,409 features for each patient were extracted, and then seven features were screened out by LASSO algorithm finally. Among the 12 significant radiomics features, we found the mean intensity of MPP was different from SPP. This might reflect different diffuse opacities or greater degree of fluid or debris affecting the airspaces leading to the diversity in airspace disease phenotypes (consolidation, ground-glass opacities, etc.) that combine varying degrees of edema and vascular and interlobular septal thickening (22, 23), so the MPP showed different irregular intensity changes, heterogeneous intensities, and range in textures from SPP. When the inflammation progresses, extensive exudation of lung tissue leads to solid pneumonia, and the early specific imaging findings of two types of inflammation were obscurated. This might also explain why the consolidation part of the pneumonia was limited in differentiation.

To date, very few studies have addressed the problem of pneumonia differentiation using radiomics. Mei et al. (24) used artificial intelligence algorithms to integrate chest CT findings with clinical symptoms, exposure history and laboratory validation to diagnose COVID-19. Wang et al. (25) combined deep learning-radiomics model to differentiate COVID-19 from non-COVID-19 viral pneumonia. These studies demonstrate the feasibility of using radiomics to identify lung inflammation. However, their study analysis showed only one kinds of pneumonia, and they did not find a significant effect of different ROI delineation methods on the results. Our approach is more pragmatic, as using only one kinds of pneumonia may introduce a selection bias and overestimate the classification accuracy. Yu et al. (26) implemented multiple network architectures to subclassify NSCLC and achieved an AUC of 0.864, which was 0.042 higher than our result. Furthermore, Wang et al. (27) conducted a similar classification task their CNN-AvgFea-Norm3-based RF method achieved an AUC of 0.856 and an accuracy of 0.820, which was 0.034 higher in AUC and 0.010 higher in accuracy compared with our classifier.

RF radiomics classifier is used widely by scientists for solving real world scale problems with limited resources (28–30). The building model and following validation results proved that RF radiomics classifier showed a good value in differentiation of mycoplasma from pneumococcal pneumonia in children, consistent with their different pathological basis. The type of pathogens in pneumonia are associated with different lymphocytes and monocytes response during the initiation (31) and hinted that radiomic model could be used as the identification method for multiple kinds of pneumonia. This provides clinicians with additional diagnostic information and promote the development of personalized precision therapy.

Although we found high diagnostic performance of radiomics model, the study still has some limitations. First, the number of patients is not large enough, external validation cannot be done due to insufficient data and the diagnostic accuracy might be overestimated. Hence, large multicenter studies are warranted to confirm the current findings. Second, the “hand-crafted” features, such as shape and texture, may not capture the full range of information contained within the images and are limited by low reproducibility. Deep learning extracts deeper and more comprehensive information directly from raw images, and has important clinical potential in disease diagnosis. Third, the study only performed two-dimensional analysis of the largest section, it may lead to inaccurate evaluation due to the influence of acquisition parameters on feature stability. Finally, the study only discuss two comment types of pneumonia in children and the others were not included.

## Conclusion

In conclusion, The RF radiomics classifier with the ROI delineation including both consolidation and surrounding halo may show potential for the differentiation of MPP and SPP, which may provide accurate differentiational diagnosis for the early and appropriate treatment.

## Data availability statement

The raw data supporting the conclusions of this article will be made available by the authors, without undue reservation.

## Ethics statement

The studies involving human participants were reviewed and approved by this study was approved by the Ethics Committee of Qilu Hospital (permit number: 2021224). Written informed consent from the participants’ legal guardian/next of kin was not required to participate in this study in accordance with the national legislation and the institutional requirements. Written informed consent was not obtained from the minor(s)’ legal guardian/next of kin for the publication of any potentially identifiable images or data included in this article.

## Author contributions

DY designed and conceptualized study, analyzed and interpreted the data, and drafted the manuscript for intellectual content. JZ, RZ, and QY analyzed and interpreted the data. DW designed and conceptualized study, analyzed and interpreted the data, and revised the manuscript for intellectual content. LZ, XH, and YQ contributed to major role in the acquisition of data. All authors were involved in drafting the manuscript or revising it and approved the final version to be published.

## Footnotes

1
http://radcloud.cn/
