# Supplementary material for: The value of CT radiomic in differentiating mycoplasma pneumoniae pneumonia from streptococcus pneumoniae pneumonia with similar consolidation in children under 5 years
Source: Front Pediatr. 2022 Sep 28;10:953399. doi: 10.3389/fped.2022.953399 (PMC9554402; doi:10.3389/fped.2022.953399)
Supplement: Supplementary file 1 [file Table_1.docx]

# Radcloud Analysis Report

## Huiying Medical Technology Co., Ltd

### 2022-6-24

#### 1. Introduction

Radiomics is an emerging field that aims at building a relevant statistical model from a large number of high-dimensional mineable features extracted from medical imaging data (possibly combined with clinical or genomic data) to assist diagnosis, prognosis and therapy monitoring.

Radiomics workflow involves: Imaging, ROI segmentation, feature extraction and analysis, then with the selected features, a statistical model is designed based on machine learning algorithms, which have to be tuned according to the clinical or biological question and to the a priori knowledge that is available.

#### 2. Materials and Methods

#### 2.1 Patients and Dataset

A total of 102 patients were included in this study. We used Radcloud (Huiying Medical Technology Co., Ltd) to manage imaging data, clinical data, and subsequent radiomics statistics analysis.

The validation dataset and training dataset were separated by random method with ratio 3:7, and the random seeds is 734

#### 2.2 Image segmentation

All these images were reviewed by two senior radiologists with 10 (reader 1) and 5 years (reader 2) experience in this field, and all the disease lesions (VOI) were delineated manually by radiologist who was blinded to the clinical information of the patients, then all contours were reviewed by the senior radiologist. If the discrepancy was ≥5%, the senior radiologist decided on the tumour borders [1].Eventually, 102 VOIs were segmented from 102 patients’ scans which were used for subject analysis.

#### 2.3 Feature extraction

A total of 1409 quantitative imaging features were extracted from CT images with Radcloud platform (http://radcloud.cn/). These features can be grouped into three groups. Group 1 (first order statistics) consisted of 126 descriptors that quantitatively delineate the distribution of voxel intensities within the CT image through commonly used and basic metrics. Group 2 (shape- and size-based features) contained 14 three-dimensional features that reflect the shape and size of the region. Calculated from grey level run-length and grey level co-occurrence texture matrices, 525 textural features that can quantify region heterogeneity differences were classified into group 3 (texture features).

#### 2.4 Feature qualification

As described above, a large number of image features may be computed. However, all these extracted features may not be useful for a particular task. Therefore, dimensionality reduction and selection of task-specific features for best performance are necessary steps. To reduce the redundant features, the feature selection methods included the variance threshold (variance threshold = 0.8), SelectKBest and the least absolute shrinkage and selection operator (LASSO) were used for this purpose. For the variance threshold method, the threshold is 0.8, so that the eigenvalues of the variance smaller than 0.8 were removed. The SelectKBest method, which belongs to a single variable feature selection method, using p value to analysis the relationship between the features and the classification results, all the features with p value smaller than 0.05 will be used. For LASSO model, L1 regularizer was used as the cost function, and the error value of cross validation is 5, and the maximum number of iterations is 1000.

#### 2.5 Statistical analysis

The statistical analysis was performed in Radcloud platform. And after feature qualification, a total of 1409 features identified were significantly correlated to this subject. Based on the selected features, there are several supervised learning classifiers available for classification analysis, which creates models that attempt to separate or predict the data with respect to an outcome or phenotype (for instance, patient outcome or response). In this study, the radiomics-based models were constructed with 6 classifiers, k-NearestNeighbor(KNN), Support Vector Machin(SVM), eXtreme Gradient Boosting(XGBoost), Random Forest (RF), Logistic Regression (LR) and Decision tree(DT), and the validation method was used to improve the effectiveness of the model.

For KNN, the parameters KNN paramsters: n_neighbors(5), weights(uniform),

For SVM, the parameters SVM paramsters: kernel(rbf), C(1), gamma(auto), class_weight(balanced), decision_function_shape(ovr), random_state(),

For XGBoost, the parameters XGBoost paramsters: Eta(0.3), max_depth(6),

For RF, the parameters RF paramsters: n_estimators(10), class_weight(None),

For LR, the parameters LR paramsters: penalty(L2), C(1), solver(liblinear), class_weight(None), multi_class(ovr), random_state(),

For DT, the parameters DT paramsters: splitter(best), criterion(gini),

To assess the predictive performance, the receiver operating characteristic (ROC) curve, namely, area under curve (AUC) were used both in training dataset and validation dataset respectively. And four indicators including P (precision = true positives / (true positives+ false positives)), R (recall = true positives / (true positives+ false negatives)), f1-score (f1-score = P*R*2/ (P+R)), support (total number in test set) to evaluate the performance of classifier in this study.

#### 3.Results

We firstly select 451 features from 1409 features using variance threshold method (Fig. 1), then with the select K best methods, we select 151 features (Fig. 2), finally, we selected 12 optimal features (Table -1) with LASSO algorithm (Fig. 3).

ROC curve analysis results were showed in Tab. 2 for training set and Tab. 3 for validation set. When training with KNN classifier, the AUC of training set were 0.907 in 2 (95% CI: 0.812 - 1.000; sensitivity 0.80 and specificity 0.81), respectively, the AUC of validation set were 0.727 in 2 (95% CI: 0.556 - 0.898; sensitivity 0.69 and specificity 0.56), respectively (Figure 4).training with SVM classifier, the AUC of training set were 0.939 in 2 (95% CI: 0.849 - 1.000; sensitivity 0.83 and specificity 0.83), respectively, the AUC of validation set were 0.797 in 2 (95% CI: 0.639 - 0.955; sensitivity 0.75 and specificity 0.69), respectively (Figure 5).training with XGBoost classifier, the AUC of training set were 1.0 in 2 (95% CI: 1.000 - 1.000; sensitivity 1.00 and specificity 1.0), respectively, the AUC of validation set were 0.785 in 2 (95% CI: 0.622 - 0.948; sensitivity 0.44 and specificity 0.75), respectively (Figure 6).training with RF classifier, the AUC of training set were 0.999 in 2 (95% CI: 0.971 - 1.000; sensitivity 0.97 and specificity 1.0), respectively, the AUC of validation set were 0.822 in 2 (95% CI: 0.684 - 0.960; sensitivity 0.81 and specificity 0.81), respectively (Figure 7).training with LR classifier, the AUC of training set were 0.888 in 2 (95% CI: 0.787 - 0.989; sensitivity 0.77 and specificity 0.78), respectively, the AUC of validation set were 0.734 in 2 (95% CI: 0.574 - 0.894; sensitivity 0.69 and specificity 0.75), respectively (Figure 8).training with DT classifier, the AUC of training set were 1.0 in 2 (95% CI: 1.000 - 1.000; sensitivity 1.00 and specificity 1.0), respectively, the AUC of validation set were 0.688 in 2 (95% CI: 0.538 - 0.838; sensitivity 0.50 and specificity 0.88), respectively (Figure 9).

We summarized these four indicators (precision, recall, f1-score, support) for classifiers in Tab. 4 and Tab. 5, respectively. When training with KNN classifier, the precision, recall, f1-score and support of training set were 0.80, 0.80, 0.80 and 35 in 2, the precision, recall, f1-score and support of validation set were 0.61, 0.69, 0.65 and 16 in 2. training with SVM classifier, the precision, recall, f1-score and support of training set were 0.83, 0.83, 0.83 and 35 in 2, the precision, recall, f1-score and support of validation set were 0.71, 0.75, 0.73 and 16 in 2. training with XGBoost classifier, the precision, recall, f1-score and support of training set were 1.00, 1.00, 1.00 and 35 in 2, the precision, recall, f1-score and support of validation set were 0.64, 0.44, 0.52 and 16 in 2. training with RF classifier, the precision, recall, f1-score and support of training set were 1.00, 0.97, 0.99 and 35 in 2, the precision, recall, f1-score and support of validation set were 0.81, 0.81, 0.81 and 16 in 2. training with LR classifier, the precision, recall, f1-score and support of training set were 0.77, 0.77, 0.77 and 35 in 2, the precision, recall, f1-score and support of validation set were 0.73, 0.69, 0.71 and 16 in 2. training with DT classifier, the precision, recall, f1-score and support of training set were 1.00, 1.00, 1.00 and 35 in 2, the precision, recall, f1-score and support of validation set were 0.80, 0.50, 0.62 and 16 in 2.

Tab. 1 Description of the selected radiomic features with their associated feature group and filter

| **Radiomic feature** | **Radiomic class** | **Filter** |
| --- | --- | --- |
| Energy | firstorder | wavelet-HHH |
| Skewness | firstorder | gradient |
| Maximum | firstorder | wavelet-HLL |
| LongRunLowGrayLevelEmphasis | glrlm | wavelet-HHH |
| Median | firstorder | lbp-2D |
| SizeZoneNonUniformity | glszm | original |
| Skewness | firstorder | wavelet-LLL |
| Maximum | firstorder | original |
| RobustMeanAbsoluteDeviation | firstorder | lbp-2D |
| RunLengthNonUniformity | glrlm | original |
| RunLengthNonUniformity | glrlm | logarithm |
| SizeZoneNonUniformity | glszm | wavelet-HLL |

Label: GLRLM=Gray Level Run Length Matrix,GLSZM=Gray-Level Size Zone Matrix

Tab. 2 ROC results with KNN, SVM, XGBoost, RF, LR and DT classifiers of training set

| **Classifiers** | **AUC** | **95% CI** | **Sensitivity** | **Specificity** |
| --- | --- | --- | --- | --- |
| KNN | 0.907 | 0.812 - 1.000 | 0.800 | 0.810 |
| SVM | 0.939 | 0.849 - 1.000 | 0.830 | 0.830 |
| XGBoost | 1.000 | 1.000 - 1.000 | 1.000 | 1.000 |
| RF | 0.999 | 0.971 - 1.000 | 0.970 | 1.000 |
| LR | 0.888 | 0.787 - 0.989 | 0.770 | 0.780 |
| DT | 1.000 | 1.000 - 1.000 | 1.000 | 1.000 |

Tab. 3 The results of AUC, 95% CI, Sensitivity, Specificity in validation cohort

| **Classifiers** | **AUC** | **95% CI** | **Sensitivity** | **Specificity** |
| --- | --- | --- | --- | --- |
| KNN | 0.727 | 0.556 - 0.898 | 0.690 | 0.560 |
| SVM | 0.797 | 0.639 - 0.955 | 0.750 | 0.690 |
| XGBoost | 0.785 | 0.622 - 0.948 | 0.440 | 0.750 |
| RF | 0.822 | 0.684 - 0.960 | 0.810 | 0.810 |
| LR | 0.734 | 0.574 - 0.894 | 0.690 | 0.750 |
| DT | 0.688 | 0.538 - 0.838 | 0.500 | 0.880 |

Tab. 4 The results of Precision, Sensitivity, F1-score,Support in training cohort

| **Indicators** | **KNN** | **SVM** | **XGBoost** | **RF** | **LR** | **DT** |
| --- | --- | --- | --- | --- | --- | --- |
| Precision | 0.800 | 0.830 | 1.000 | 1.000 | 0.770 | 1.000 |
| Sensitivity | 0.800 | 0.830 | 1.000 | 0.970 | 0.770 | 1.000 |
| F1-score | 0.800 | 0.830 | 1.000 | 0.990 | 0.770 | 1.000 |
| Support | 35 | 35 | 35 | 35 | 35 | 35 |

Tab. 5 The results of four indicators -Precision, Sensitivity, F1-score, Support in validation set

| **Indicators** | **KNN** | **SVM** | **XGBoost** | **RF** | **LR** | **DT** |
| --- | --- | --- | --- | --- | --- | --- |
| Precision | 0.610 | 0.710 | 0.640 | 0.810 | 0.730 | 0.800 |
| Sensitivity | 0.690 | 0.750 | 0.440 | 0.810 | 0.690 | 0.500 |
| F1-score | 0.650 | 0.730 | 0.520 | 0.810 | 0.710 | 0.620 |
| Support | 16 | 16 | 16 | 16 | 16 | 16 |


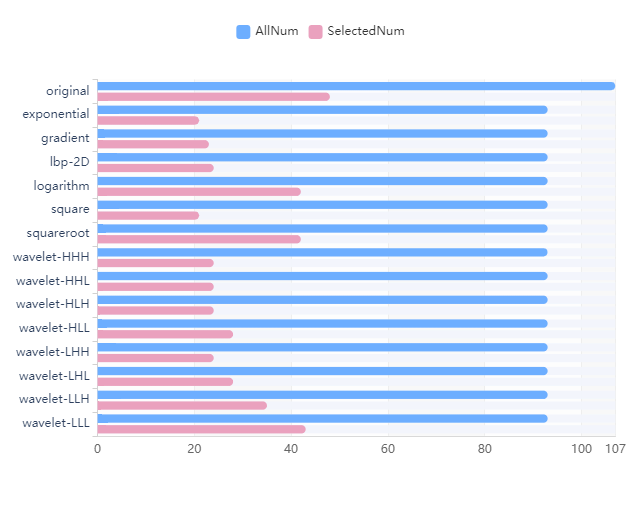


wavelet-LLL
AllNum: 93
SelectedNum: 43

Fig. 1. Variance threshold on feature select. We used variance threshold methods to select radiomics features (variance threshold = 0.8), we selected 451 features from 1409 features.


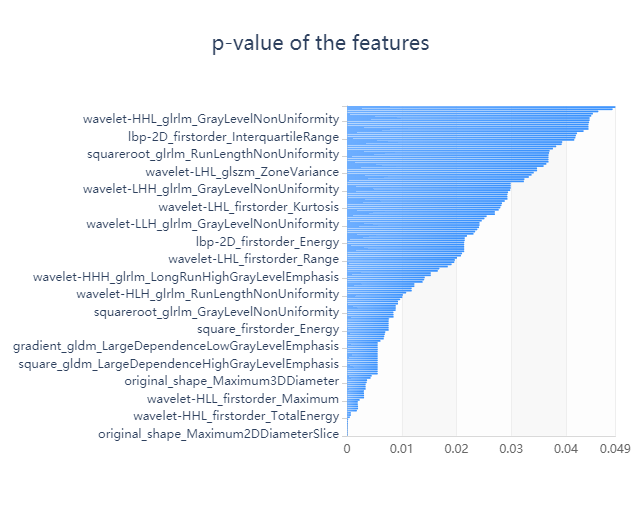


Fig. 2. Select K best on feature select. We used Select K best methods to further select radiomics features, we selected 151 features.

#### Lasso parameters: cv(5) and max_iter(1000)


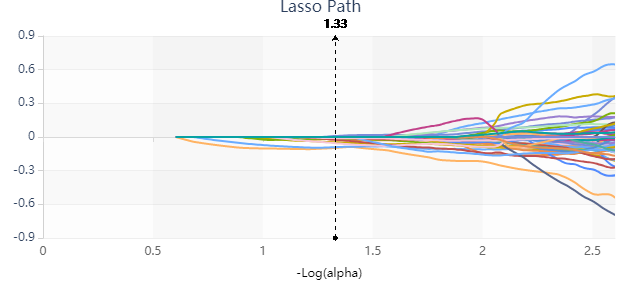


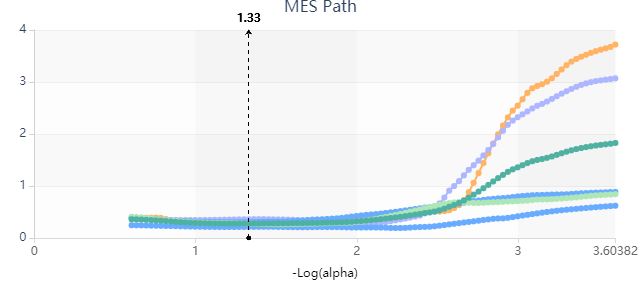


1.91
0: 0.3467399260321667
1: 0.28290959324847187
2: 0.20762425560524542
3: 0.38592908389844915
4: 0.29196482965844517
average: 0.3030335376885557


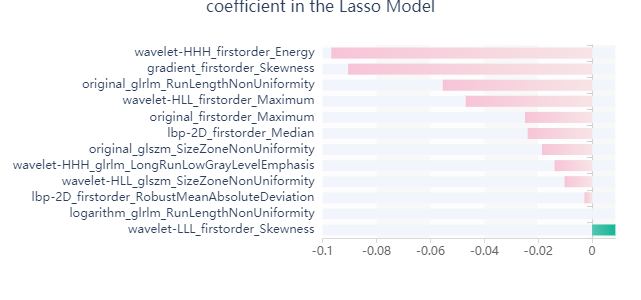


wavelet-HHH_glrlm_LongRunLowGrayLevelEmphasis
-0.0139

Fig. 3. Lasso althorithm on feature select. (a) Laso path; (b) MSE path; (c) coefficients in Lass model. Using Lasso model, 12 features which are correspond to the optimal alpha value were selected.

KNN paramsters: n_neighbors(5), weights(uniform),


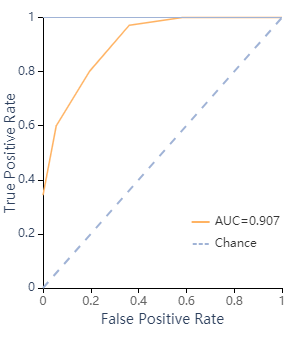


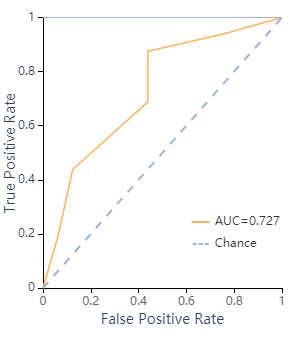


Fig. 4. ROC curves of KNN methods to classification. (a) ROC curve of training set, the AUC were 0.907 in 2 (sensitivity and specificity were 0.80 and {"2": 0.81}) respectively; (b) ROC curve of validation set, the AUC were 0.727 in 2 (sensitivity and specificity were 0.69 and {"2": 0.56}) respectively.

SVM paramsters: kernel(rbf), C(1), gamma(auto), class_weight(balanced), decision_function_shape(ovr), random_state(),


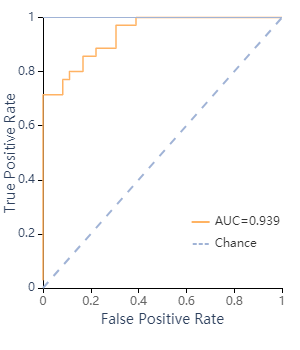


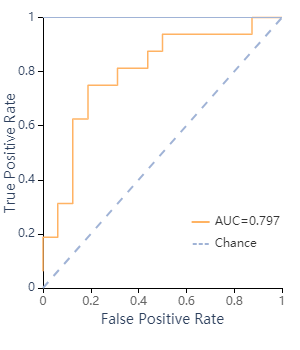


Fig. 5. ROC curves of SVM methods to classification. (a) ROC curve of training set, the AUC were 0.939 in 2 (sensitivity and specificity were 0.83 and {"2": 0.83}) respectively; (b) ROC curve of validation set, the AUC were 0.797 in 2 (sensitivity and specificity were 0.75 and {"2": 0.69}) respectively.

XGBoost paramsters: Eta(0.3), max_depth(6),


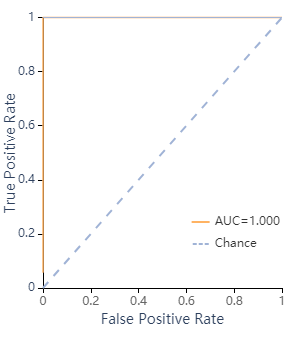


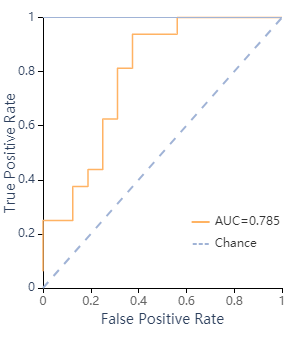


Fig. 6. ROC curves of XGBoost methods to classification. (a) ROC curve of training set, the AUC were 1.0 in 2 (sensitivity and specificity were 1.00 and {"2": 1.0}) respectively; (b) ROC curve of validation set, the AUC were 0.785 in 2 (sensitivity and specificity were 0.44 and {"2": 0.75}) respectively.

RF paramsters: n_estimators(10), class_weight(None),


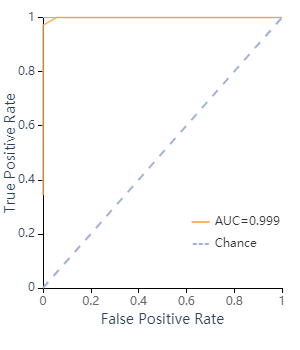


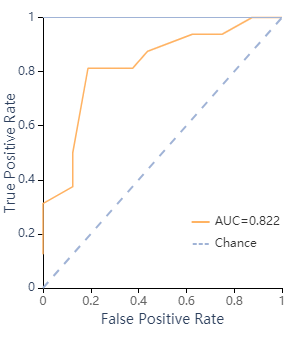


Fig. 7. ROC curves of RF methods to classification. (a) ROC curve of training set, the AUC were 0.999 in 2 (sensitivity and specificity were 0.97 and {"2": 1.0}) respectively; (b) ROC curve of validation set, the AUC were 0.822 in 2 (sensitivity and specificity were 0.81 and {"2": 0.81}) respectively.

LR paramsters: penalty(L2), C(1), solver(liblinear), class_weight(None), multi_class(ovr), random_state(),


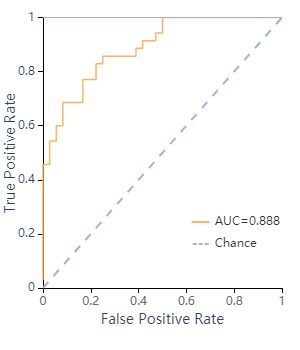


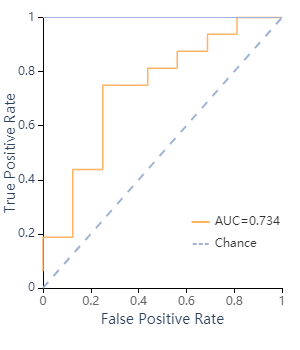


Fig. 8. ROC curves of LR methods to classification. (a) ROC curve of training set, the AUC were 0.888 in 2 (sensitivity and specificity were 0.77 and {"2": 0.78}) respectively; (b) ROC curve of validation set, the AUC were 0.734 in 2 (sensitivity and specificity were 0.69 and {"2": 0.75}) respectively.

DT paramsters: splitter(best), criterion(gini),


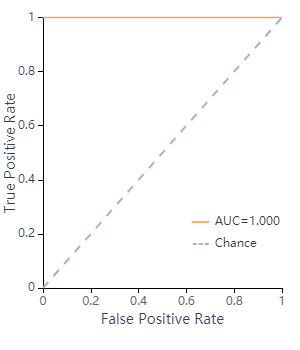


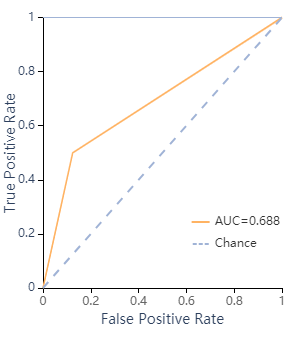


Fig. 9. ROC curves of DT methods to classification. (a) ROC curve of training set, the AUC were 1.0 in 2 (sensitivity and specificity were 1.00 and {"2": 1.0}) respectively; (b) ROC curve of validation set, the AUC were 0.688 in 2 (sensitivity and specificity were 0.50 and {"2": 0.88}) respectively.

#### 4.References

1. Lambin P, Rios-Velazquez E, Leijenaar R, et al. Radiomics: extracting more information from medical images using advanced feature analysis. European journal of cancer, 2012, 48(4): 441-446.

2. Kumar V, Gu Y, Basu S, et al. Radiomics: the process and the challenges.[J]. Magnetic Resonance Imaging, 2012, 30(9): 1234-1248.

3. Gillies R J, Kinahan P E, Hricak H, et al. Radiomics: Images Are More than Pictures, They Are Data[J]. Radiology, 2016, 278(2): 563-577.

4. Wang YY, Zhang T, Li SWet al. Mapping p53 mutations in low-grade glioma: a voxel-based neuroimaging analysis. AJNR Am J Neuroradiol, 2015, 36:70–76.

#### Appendix

#### Radcloud cloud platform

Radiomics cloud platform uses cloud computing, big data analysis and machine learning algorithms to manage DICOM imaging data, medical check-up reports and clinical information on cloud platforms of hospitals. Anytime, anywhere, with only one click, research fellows can use the radiomics cloud platform to process and analyze data.

#### Research Project Management

Management of user access on data sharing among research project teams is now possible; separation of individual’s projects and cooperation projects is already a reality. This platform supports cloud management, remote cooperation and group sharing.

#### Multi-center Cooperation Management

Based on mixed cloud construction and user access management, this platform supports group visiting and cross-department, inter-agency cooperation.

#### Radiomics Statistics Analysis

More than 1,000 Ml eigenvalue for analysis, combined with machine learning, we have the one-dick operation for analysts.

#### Imaging and Clinical Data Management

Supports auto batch upload of imaging data, simultaneous data cleansing, input of dinical and pathological data. Also supports precise searching of Ml.
